# Multiscale physics-based *in silico* modelling of nanocarrier-assisted intravascular drug delivery

**DOI:** 10.3389/fddev.2024.1362660

**Published:** 2024-03-04

**Authors:** Nicolae-Viorel Buchete, Iwona Cicha, Sutapa Dutta, Panagiotis Neofytou

**Affiliations:** ^1^ School of Physics, University College Dublin, Dublin, Ireland; ^2^ Institute for Discovery, University College Dublin, Dublin, Ireland; ^3^ ENT-Department, Section of Experimental Oncology und Nanomedicine (SEON), Universitätsklinikum Erlangen, Erlangen, Germany; ^4^ Thermal Hydraulics and Multiphase Flow Laboratory, Institute of Nuclear & Radiology Sciences and Technology, Energy & Safety, National Centre for Scientific Research “Demokritos”, Athens, Greece

**Keywords:** drug delivery nanocarriers, atomistic molecular modelling, coarse-grained molecular modeling, nanoparticle protein corona, fluid-nanocarrier interaction modelling, computational fluid-particle dynamics

## Abstract

A rational design of drug nanocarriers supported by *in silico* modelling tools can improve the efficacy of nanosystem-based intravascular drug delivery (IVDD). Computational model development stems from the vision of replacing conventional (pre)clinical trials with advanced simulations and applies to the development of more efficient nanocarriers for intravascular therapies. To establish a standardized framework for *in silico* preclinical trials, it is necessary to include *in silico* tools that can model each experimental stage of a preclinical trial for a respective nanocarrier system and give accurate and verifiable results. This review paper highlights the status of intravascular drug delivery supported by nanocarriers and discusses the modelling stages of a physics-based multiscale modelling framework that should be developed, validated and exploited to address the need for an effective preclinical assessment of nanocarriers for IVDD.

## 1 Introduction to nanoparticle-based drug delivery

Nanoparticles represent an innovative platform, that can be adapted to serve both therapy and the diagnosis of various human diseases. Although multiple delivery routes are possible, the majority of the clinically-relevant nanocarriers, such as anti-cancer and anti-inflammatory nanoparticulate drugs, require intravascular administration. However, the first-generation injectable drug carriers have often been plagued by suboptimal efficacy as a result of rapid clearance by the reticuloendothelial system (RES) (Alexis et al., 2008), or of an inability to reach the target tissue in effective dosage (Chen et al., 2006; Kennel et al., 2008; Moros et al., 2013). Consequently, a rational design of drug carriers supported by modeling tools is expected to improve the efficacy of nanodrugs, by enabling the delivery of a sufficient payload of therapeutics to the diseased regions, limiting their cyto- and genotoxicity, and preventing their unspecific clearance by the RES.

### 1.1 Nanoparticle types

Nanocapsules or nanoparticles composed of different natural or synthetic materials are gaining attention as potential intravascular drug delivery (IVDD) systems. The commonly reported drug-carrier nanosystems have been the topic of multiple reviews in the previous years (Mitchell et al., 2021; Joudeh and Linke, 2022) and their basic characteristics are briefly outlined below.

#### 1.1.1 Lipid and polymer-based nanoparticles

*Liposomes* consist of a lipid bilayer containing amphipathic phospholipids (primarily phosphatidylcholine) that enclose an interior aqueous space (Puri et al., 2009). As they are commonly synthesized from natural phospholipids, their toxicity is relatively low (Huwyler et al., 2008; Puri et al., 2009; Balazs and Godbey, 2011; Muller et al., 2011), but there are little data concerning the effects of increased shear forces on their integrity and they are easily cleared from the circulation by RES. To improve the stability and circulation time of liposomes, the head groups of phospholipids can be conjugated with polyethylene glycol (PEG), resulting in so-called stealth liposomes (Almer et al., 2013). Functionalization with maleimide as the PEG end-group allows conjugation of antibodies or other targeting ligands on their surface (van der Meel et al., 2013).

*Lipid-core nanoparticles* (LCNPs) are composed of a lipid kernel stabilized by a single layer of surfactant shell, which commonly contains a mixture of phospholipids, PEGylated surfactants, or amphiphilic saccharides (Gravier et al., 2011; Devel et al., 2019). Based on the state of their lipidic core components, LCNPs are classified in three categories: liquid lipid NPs (including lipid nanoemulsions and lipid nanocapsules), solid lipid NPs, and nanostructured lipid nanocarriers (Graván et al., 2023). Their size is easily adjustable and lipid composition is similar to those of low-density lipoproteins, enabling prolonged blood circulation and accumulation in atherosclerotic lesions (Devel et al., 2019).

*Polymer nanoparticles* are composed of synthetic or natural polymers, most commonly poly (lactic-*co*-glycolic acid) (PLGA), poly (lactic acid), poly (caprolactone), poly (alkylcyanoacrylates), or chitosan (Lai et al., 2014). The polymer core of the nanoparticles can be covalently cross-linked with the coating, forming a biocompatible hydrophilic shell. Dextrans, stable glucose polymers, that contain functional groups for derivatization (Sun and Mao, 2012), are commonly used to improve colloidal stability and biocompatibility of such particles. Their tunable surface properties enable easy grafting of functional groups for conjugation of drugs or targeting ligands (Bachelet et al., 2009; Rouzet et al., 2011).

#### 1.1.2 Inorganic nanoparticle systems

*Gold nanoparticles* are composed of a dielectric core of silica coated with a metallic layer of gold, or consist of gold core encircled by an organic monolayer, and are tunable to various sizes and forms (Menon et al., 2013). Owing to their optical properties, these particles can be used for e.g., for optical imaging, or photothermal ablation therapy, as well as drug carriers (Lv et al., 2022). However, the important concern related to gold particles is their potential cytotoxicity and a slow elimination resulting in a long-term persistence within many organs (Xia et al., 2016).

*Silver nanoparticles* usually consist of silver cores stabilized with silica or sodium citrate and their properties range from broad-spectrum antimicrobial efficacy against bacteria, fungi and viruses (Hancharova et al., 2024) to photosensitizing effects (Singh et al., 2024). Consequently, these particles are tested in multiple *in vivo* applications including wound care, imaging, cancer therapy or tissue engineering, but their long-term safety and biocompatibility remain to be studied.

*Superparamagnetic iron oxide nanoparticles (SPIONs)* consist of iron oxide core and a shell that is usually composed of organic materials such as fatty acids, polysaccharides, or polymers, aiming to ensure their colloidal stability, biocompatibility and functionalization with drugs or targeting moieties (Tassa et al., 2011; Wahajuddin and Arora, 2012). Owing to their magnetic properties, SPIONs are used as intravascular contrast agents for magnetic resonance imaging (Tang et al., 2009; Sadat et al., 2013; Liu et al., 2014), or for hyperthermia-therapy of cancer (Hayashi et al., 2013).

*Quantum dots* (QDs) - nanoscale semiconductor crystals with sizes 2–10 nm - are most commonly composed of binary compounds containing elements such as Cd, Pb, Hg, In, Se (Ji et al., 2014). The fluorescence bands of QDs are dependent on their composition, size and shell thickness (Marukhyan and Gasparyan, 2017). Besides serving as imaging tools, QDs have great potential as theranostics platforms for simultaneous imaging and therapy.

*Carbon-based nanomaterials* characterized by exceptional mechanical, electrical, and thermal properties include fullerenes, graphene quantum dots (GQDs), graphene oxide (GO) and carbon nanotubes (CNT), which were recently reviewed in Malode et al. (2024). Their potential biomedical applications include, among others, biosensing, hyperthermia, drug delivery and tissue engineering (AbouAitah et al., 2023; Sharma et al., 2024), as well as improving the electric conductivity of cardiac patches (Ren et al., 2017).

The above-listed nanomaterials can be combined in order to tailor the characteristics of the final nanosystem, e.g., biocompatibility, colloidal stability, specific charge, size or imaging properties. Given adequate safety and effective drug delivery, such hybrid nanosystems with adjustable characteristics could improve the future therapeutic success of intravascular drug carriers.

### 1.2 Nanoparticle loading with drugs

The first FDA-approved nanodrug was a PEGylated liposomal formulation of doxorubicin (Doxil^®^), indicated for the treatment of several types of cancer [reviewed in Barenholz (2012)]. Following this, multiple research groups focused on the development of nanocarriers for drug delivery. Several of the reported carriers and their cargo are briefly highlighted below.

Chemotherapeutics, including doxorubicin, are commonly used in the development of nanosystems aiming at intravascular drug delivery to cancer. Research is ongoing on further improvement of doxorubicin-loaded liposomal nanocapsules alone (Song P. P. et al., 2022; Shim et al., 2022), or in combination with other anti-cancer drugs (Gabizon et al., 2021). Also, liposomal paclitaxel has already entered clinical practice (Zhang W. W. et al., 2022). The development of PEGylated liposomes loaded with oxaliplatin and paclitaxel has been recently reported (Xie et al., 2023) as well as liposomes containing a co-load of hesperetin and cisplatin (Wang X. et al., 2023). Lipid-based nanoparticles can also serve as carriers of biologics, such as nucleic acids (Gilbert et al., 2024). In cancer immunotherapy, ionizable LNPs were developed as carriers of mRNA (Billingsley et al., 2023) or plasmid DNA (Prazeres et al., 2023) to induce chimeric antigen receptor (CAR) expression in T cells.

Polymer nanoparticles as carriers of several anti-cancer agents, including doxorubicin and paclitaxel have been reported (Danhier et al., 2012; Geary and Salem, 2014; Zhang D. et al., 2022; Almoustafa et al., 2023; Miraghaie et al., 2023). Recently, a combination chemo-immunotherapy for cancer using lipid polymer nanocomplexes based on PLGA and 1, 2-distearoyl-sn-glycero-3-phosphoethanolamine (DSPE)-PEG was also proposed. The developed nanosytem served as a carrier of doxorubicin, and as a checkpoint inhibiting peptide against programmed death-ligand 1 (PD-L1) (Zhang N. et al., 2022).

Gold nanoparticles have been utilized for experimental delivery of anti-cancer drugs, such as gemcitabine (Kudgus et al., 2013) doxorubicin (Zhang et al., 2015; Morshed et al., 2016), as well as cisplatin (Quinones et al., 2023), [reviewed in Moreno-Alcantar et al. (2023)]. Cisplatin delivery system based on silver nanoparticles coated with an alginate hydrogel shell was also reported (Maher et al., 2023). Cai et al. reported the development of ultrasmall pH-responsive ZnO QDs functionalized with PEG that were used as carriers of doxorubicin (Cai et al., 2016). QDs were also loaded with small interfering RNA targeting Bcl-2 and different anticancer drugs (carboplatin, paclitaxel, and doxorubicin) for combined therapy of lung cancer (Li et al., 2016).

Similarly, many research groups reported SPION-based nanosysems for drug delivery of chemotherapeutic agents, including doxorubicin (Hernandes et al., 2023; Markhulia et al., 2023), cisplatin (Unterweger et al., 2014; Hernandez et al., 2023), mitoxantrone (Tietze et al., 2013), among others [reviewed in Vangijzegem et al. (2023)]. Dual drug-loading approach was also reported using SPIONs functionalized with anti-angiogenic peptide and loaded with paclitaxel (Ngema et al., 2023).

Further, theranostic nanosystems composed of two or more types of materials have been developed, combining magnetic particles with polymers (Amiryaghoubi et al., 2022) or liposomes (Shetake et al., 2022) for delivery of doxorubicin to cancer. An interesting approach to metastatic breast cancers was recently reported by Hasannia et al., who developed nanovessicles based on the poly (L-glutamic acid) and termed peptosomes, that were loaded with magnetic particles and doxorubicin (Hasannia et al., 2023).

However, compared to the field of oncology, fewer research groups pursue the development of nanosystems for cardiovascular drug delivery. Interestingly, nearly all particle types mentioned above have been employed as experimental drug carriers for various cardiovascular disorders [see a recent review by Luo et al. (2023)]. Some of the main recent examples are highlighted below.

Liposomes have been used as carriers of anti-atherosclerotic drugs including statins (Li et al., 2019; Wu et al., 2021), PCSK9 inhibitor evolocumab (Li et al., 2023), or docosahexaenoic acid (Chong et al., 2023), as well as anti-inflammatory agents such as prednisolone (van der Valk et al., 2015), methotrexate (Di Francesco et al., 2020), or micro RNA (miR-146a) (Ho et al., 2023). Delivery of nucleic acids has furthermore been employed to reduce plasma cholesterol levels using liver-specific ionizable LNPs loaded with small interfering RNA silencing Pcsk9 gene (Huang et al., 2023) and to prevent cardiac fibrosis in heart failure using LNPs carrying modified mRNA encoding the antifibrotic CAR to T lymphocytes (Rurik et al., 2022). In general, lipid-based nanosystems, including liposomes, solid lipid particles as well as reconstituted high-density lipoprotein (HDL) nanoparticles display increased affinity for macrophages, or dendritric cells (Courant et al., 2017; Kornmueller et al., 2019), and accumulate in atherosclerotic plaques, which translates in improved efficacy *versus* free drugs (Wang et al., 2018).

Polymer particles have been frequently used as nanocarriers for the treatment of cardiovascular diseases (Mura and Couvreur, 2012; Nenna et al., 2021). In particular nanosystems based on PLGA, the most common biodegradable polymer approved for use in humans, have been tested in animal models as drug carriers for tissue plasminogen activator (Korin et al., 2012), streptokinase (Hasanpour et al., 2021), superoxide dismutase (Yun et al., 2013), bevacizumab (De Negri Atanasio et al., 2022), pioglitazone (Nakashiro et al., 2016; Todaro et al., 2022; Groner et al., 2023), senolytic drug ABT263 (Lee et al., 2021), as well as combined gene delivery of VEGF and NGF (Chen Y. et al., 2022) and microRNAs that effectively regulate Nrf2 against myocardial ischemia-reperfusion injury (Wang et al., 2022).

Polymer nanocarriers are also very commonly used for delivery of statins (reviewed in (Montelione et al., 2023), including simvastatin (Alaarg et al., 2017), pitavastatin (Nagaoka et al., 2015; Katsuki et al., 2022), or atorvastatin (Liu et al., 2023). The latter study compared the drug loading and efficacy of polymeric (PLGA and poly-vinyl alcohol) carries with PLGS-lipid hybrid particles. PLGA-based nanosystem was furthermore used to develop reactive oxygen species (ROS)-sensitive complexes for co-delivery of siRNA against vascular cell adhesion molecule-1 (VCAM-1) and dexamethasone in a rat model of myocardial ischemia-reperfusion (Hou et al., 2022).

Compared to polymer or lipid-based nanosystems, metal-based nanoparticles have more commonly been used as imaging agents rather than drug carriers. However, their theranostic potential is increasingly getting acknowledged and the numbers of reports describing new developments of metal-based carriers is growing (Younis et al., 2021). SPIONs have been approved as clinical contrast agents for MRI (Sadat et al., 2013; Florian et al., 2014; Nguyen et al., 2019; Zheng et al., 2019), but are less often used for cardiovascular drug delivery. Of note, SPION-based nanosystems have been developed by different groups as a platform for delivery of thrombolytic drugs (e.g., tPA, streptokinase), either alone (Ma et al., 2009; Tadayon et al., 2015), or in combination with PLGA (Chen et al., 2020) or PLGA and chitosan (Zhou et al., 2014). The delivery of dexamethasone-loaded SPIONs to atherosclerotic plaques (Matuszak et al., 2018) and ischemic brain regions (Lu et al., 2021) was also reported. The initial *in vitro* evaluation of clinically approved SPION formulation (ferumoxytol) functionalized with vascular endothelial growth factor (VEGF) for potential future use in patients with myocardial infarction showed their positive effect on cell survival and growth (Bietenbeck et al., 2019).

Another type of nanosystem relatively rarely used for cardiovascular drug delivery is based on gold nanoparticles. Clinically approved inotropic agent levosimendan loaded on gold nanoparticles was previously tested in a rat model of heart failure (Spivak et al., 2013). Gold nanoparticles in combination with highly branched poly (amide amine) (PAMAM) dendrimers were also used as carriers of gastrodin, a natural neuroprotective phenolic glycoside, in a model of cerebral ischemia-reperfusion injury (Huang et al., 2022). For thrombotic disorders, mesoporous silica-coated gold nanorods conjugated with near-infrared fluorescent dye have been developed as theranostic carriers of urokinase-type plasminogen activator and algae-derived P-selectin specific anticoagulant fucoidan (Chang et al., 2021).

Similarly, graphene quantum dot-based drug delivery system to atherosclerosis has been developed only recently. Loaded with microRNA223 linked by disulfide bonds, it has been grafted onto monocyte membranes to ensure delivery on the cells transmigrating to the interior of the plaque in ApoE-deficient mice (Liu F. et al., 2019).

### 1.3 Nanoparticle targeting and interactions with target tissues/cells

The parenterally-administered nanosystems for drug delivery should be able to achieve an increased circulation half-life to avoid scavenging by RES (Haroon et al., 2022), and a high margination rate in bloodstream to allow enhanced interactions with endothelial cells in the target region (Decuzzi et al., 2009).

*Passive targeting* can be used to deliver nanotherapeutics to the diseased regions, but its efficacy is limited to the microvascular beds where the blood-tissue barrier is compromised, e.g., due to cancer or inflammation (Claesson-Welsh et al., 2021). The hypoxic center of the growing tumor induces angiogenic effect on the surrounding vasculature leading to neovascularization. Due to the conditions in tumor microenvironment, including high interstitial pressure and lack of proper cellular and connective tissue support of the forming vessels, the tumor vasculature is malformed, immature and leaky (Nakamura et al., 2016). The increased permeability of tumor microvessels enables entry of the macromolecules and nanoparticles into the tumor tissue. At the same time, the stromal pressure prevents the formation of functional lymphatic vessels draining the tumor, thus enhancing the retention of molecules and particles in the tumor tissue. The enhanced permeability and retention (EPR) effect can therefore improve the accumulation of chemotherapeutic drugs via compromised endothelial barrier (Wu, 2021). However, despite of it, only about 1% of circulating nanosystems reach the tumor tissue (Wilhelm et al., 2016). EPR effect is also present in advanced atherosclerotic lesions, as the immature vasa vasorum of those plaques resemble the leaky tumor vessels. Another biological mechanism of passive targeting to atherosclerosis utilizes the increased permeability of luminal endothelium for accumulation of lipid-/or lipoprotein-based particles in the plaques. In this context, especially HDL-mimetic particles can be employed as drug carriers targeting atherosclerotic plaques (Sanchez-Gaytan et al., 2015). However, as passive targeting offers limited efficacy, more specific or *active targeting* approaches are required to enable an increased accumulation of particles in the diseased tissues or vascular beds. These are realized by means of molecular targeting to a specific peptide or receptor expressed on the target cells, or by magnetic targeting method to remotely control the accumulation of drugs in the diseased region.

#### 1.3.1 Active targeting of cancer

Active targeting of tumor cells is commonly realized by functionalization of nanoparticle surface with specific antibodies or ligands, e.g., hyaluronic acid (HA), a ligand of tumor-associated receptor CD44; folic acid, a ligand of folate receptor; transferrin, a ligand of transferrin receptor; RGD peptide, a ligand of integrins, or antibodies inhibiting epidermal growth factor receptor (EGFR), also known as HER1 (human epidermal receptor 1) [reviewed in Hong et al. (2023)]. Furthermore, the active targeting of paclitaxel using SPIONs functionalized with anti-VEGF peptide, HRH, has recently been reported (Ngema et al., 2023).

#### 1.3.2 Active targeting of cardiovascular disorders

**FIGURE 1:**
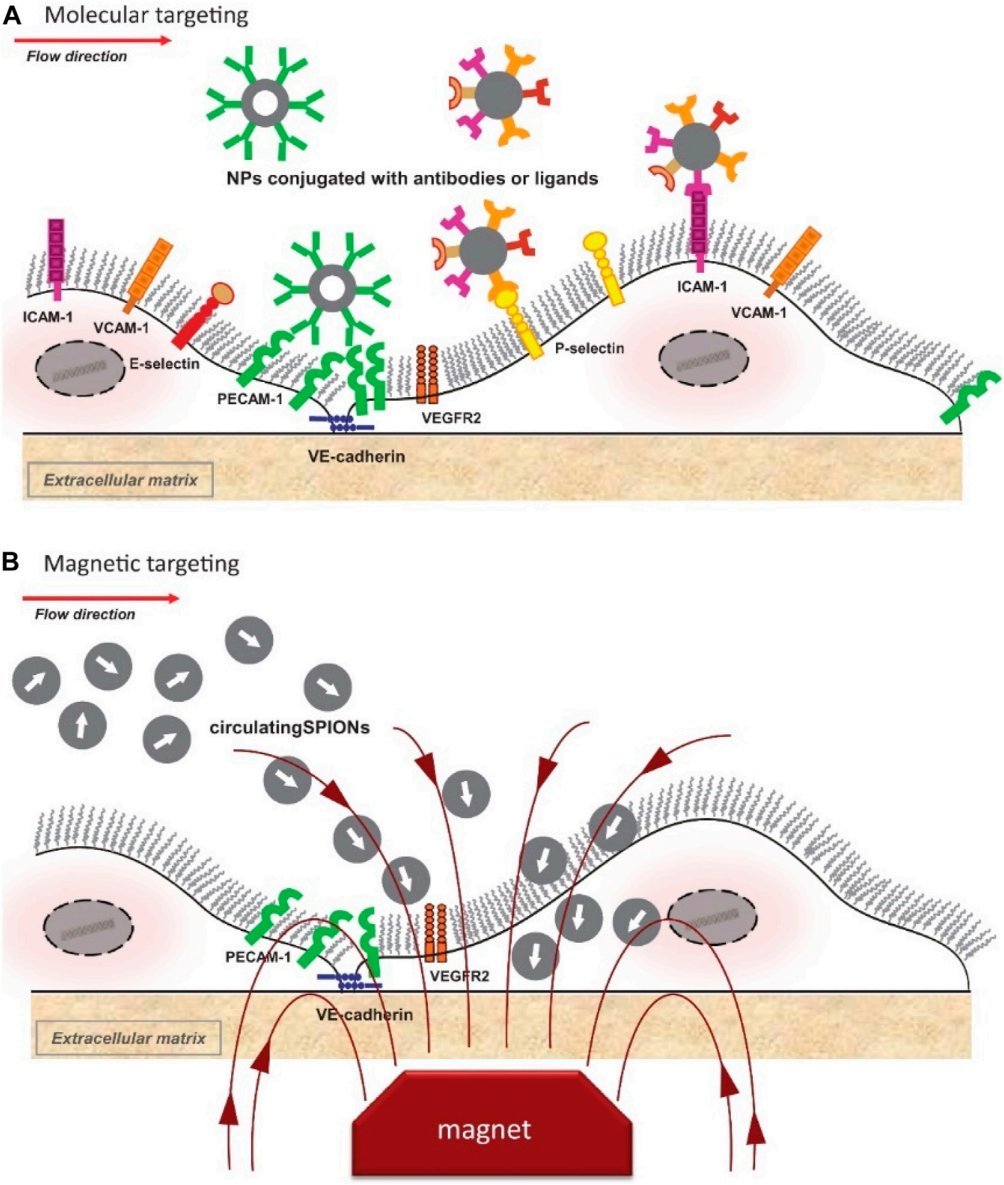
Schematic representation of active targeting of endothelial cells with intravascular nanoparticles. **(A)** Molecular-based-targeting; **(B)** Magnetic-based targeting. Vascular endothelial cadherin (VE-cadherin), vascular endothelial growth factor receptor 2 (VEGFR2), and other important biomolecular components, including antibodies and ligands, are also illustrated schematically. Reprinted with permission of IOS Press from publication (Cicha, 2016), Copyright 2016, available at IOS Press through: https://doi.org/10.3233/jcb-15020.

Previous experimental studies *in vitro* models indicated that accumulation of nanocarriers under flow conditions typical for medium and large human arteries may not be sufficient due to the hemodynamic conditions preventing nanoparticle interactions with cells (Yang et al., 2011; Yang et al., 2013). *In vivo*, untargeted drug delivery into endothelium strongly depends different physiological hemodynamic patterns (capillaries vs. arterioles/arteries) or pathological conditions (e.g., inflammation). Extensive efforts are therefore focused on identifying targeting approaches that could enhance particle accumulation at the disease-specific regions and binding of nanoparticles to vascular endothelium (Figure 1).

#### 1.3.3 Molecular targeting of vascular cells

Endothelial cells: Endothelial activation markers, such as adhesion molecules have been tested as molecular targets *in vitro* and *in vivo* already in the 2000s [reviewed in Cicha (2016)]. Ligands or antibodies against VCAM-1; (Yang et al., 2013; Dosta et al., 2021; Reyes-Esteves et al., 2023), intercellular adhesion molecule-1 [ICAM-1; (Bhowmick et al., 2012; Zukerman et al., 2020)], platelet-endothelial cell adhesion molecule-1 [PECAM-1 (Han et al., 2012; de Castro Leao et al., 2021)], as well as E-selectin (Ma et al., 2016; Inyang et al., 2020) were used to functionalize different types of nanoparticles and enhance their internalization by endothelial cells. As an example, functionalization of cationic lipoparticles containing anti-miR-712 with VCAM-1 ligand enabled their specific accumulation in inflamed mouse endothelium and led to effective inhibition atherosclerotic plaque formation (Kheirolomoom et al., 2015). Furthermore, nanoparticles functionalized with Arg-Gly-Asp (RGD) peptide, a ligand of α_v_β_3_ integrin, which is highly expressed on the luminal surface of activated endothelial cells and mediates angiogenesis, have been investigated as carriers for targeted delivery of drugs to activated endothelial cells (Martinez-Jothar et al., 2020; De Negri Atanasio et al., 2022). Another very effective targeting approach involves platelet-mimicry by conjugation of glycoproteins (Kona et al., 2012) or platelet membrane cloaking (Hu et al., 2015), which enables selective binding to endothelial cells under arterial flow conditions. Collectively, these results indicate that molecular targeting can significantly improve endothelial interactions with nanocarriers *in vivo* and allow enhanced drug delivery even in large vessels under physiologic flow conditions.

Macrophages: Targeting of macrophages in atherosclerotic plaques is commonly realized by functionalization of the nanocarriers’ surface with a ligand of scavenger receptor-AI (Ye M. et al., 2019; Bai et al., 2022; Zhu et al., 2023), or a ligand of class II scavenger receptor, CD36 (Wang Q. et al., 2021), which are highly expressed on in macrophages and foam cells. Furthermore, owing to a high expression of CD44 in inflammatory macrophages, HA is commonly used to target drug-loaded nanocarriers towards atherosclerotic plaque (Song K. et al., 2022; He et al., 2023). Nanoparticles conjugated with antibody against CD11b expressed on monocytes have also been shown to effectively target the ischemic region of the heart and improve outcomes in a mouse model of MI (Li et al., 2022). Further approaches to macrophage targeting in atherosclerosis have been recently reviewed in Chen W. et al. (2022).

Platelets: Markers of activated platelets, such as P-selectin or integrin αIIbβ3, have been commonly used to target drug carriers to thrombotic regions. For this, the surface of nanocarriers has been functionalized with e.g., fucoidan, an algae-derived P-selectin ligand (Chang et al., 2021; Zhang et al., 2021; Wang Y. et al., 2023), or platelet-binding peptide (CGSSSGRGDSPA) that binds to integrin αIIbβ3 (Sun et al., 2020; Sun et al., 2023). Other recently tested approaches enabling targeting of blood clots forming in the vasculature include fibrin targeting with fibrin-binding peptide (cyclo-AC-Y (DGI)C(HPr)YGLCYIQGK-Am) (Sun et al., 2023), or CREKA (Zhao et al., 2020), as well as surface-decoration with vWF-binding peptides (TRYLRIHPQSWVHQI) and collagen-binding peptides (GPO) via conjugation to maleimide-terminated lipid molecules and subsequent incorporation into liposomal nanocarriers (Girish et al., 2022).

#### 1.3.4 Magnetic targeting

In contrast to molecular targeting, which involves functionalization of nanocarriers with specific target-binding molecules, magnetic drug targeting utilizes the intrinsic properties of SPIONs and an external magnetic field to direct and accumulate drug-loaded carriers at the diseased regions (Figure 1). Magnetic targeting has a very high efficacy, but low specificity towards particular cell-types. Due to the specific hemodynamic conditions, this approach is easier to realize in tumor tissues or microvessels, compared to arterial circulation, where magnetic forces must overcome the increased flow rate and pressure.

In cancer therapies, magnetic targeting enables the efficient drug delivery resulting in improved therapeutic outcome [reviewed in Tietze et al. (2015)]. Delivery of doxorubicin in mouse models of hepatoma (Chao et al., 2012), Ehrlich carcinoma (Elbialy et al., 2015), or ovarian cancer (Yu et al., 2015), as well as mitoxantrone in a rabbit tumor model (Tietze et al., 2013), confirmed that active targeting using the magnetic field enhances the specific drug delivery to the tumor vasculature and increases its therapeutic efficacy.

The magnetic properties of SPIONs were also shown to allow magnetic drug targeting in arteries (Chen et al., 2008). Dexamethasone-loaded SPIONs were magnetically targeted to the regions of balloon injury in abdominal aorta and to advanced atherosclerotic plaques in rabbits (Matuszak et al., 2018). In a rat model of myocardial ischemia, magnetic nanobeads carrying VEGF gene were targeted to injured hearts using epicardial magnet (Zhang et al., 2012). Magnetic targeting found a particularly promising application in the treatment of thrombosis, where systemic administration of thrombolytic drugs bears a risk of hemorrhagic complications and local drug delivery is limited by occlusive thrombi. In this context, the delivery of SPIONs loaded with tPA (Ma et al., 2009; Liu C. H. et al., 2019; Wang L. et al., 2021) has a great potential for rapid and localized thrombolysis with minimized side effects.

#### 1.3.5 Unspecific off-target effects and clearance

While the targeted delivery of nanocarrier-bound drugs can enhance their local accumulation in the diseased tissues thus improving therapeutic efficacy and minimizing systemic side effects, the extended circulation time of nanocarriers, as well as multiple degradation products, may result in cytotoxicity (Kumar and Dhawan, 2013), or immunogenicity (Zolnik et al., 2010). Apart from systemic toxicity, the escape of the drug/carrier from the target tissue is expected to reduce its efficacy, as the particle-bound drugs will eventually undergo clearance in liver, kidney, or spleen.

It is therefore important to develop tools supporting the design of drug carriers and their early validation before entering the extensive and costly *in vivo* experiments. For this, modeling of ligand-receptor, nanocarrier-blood protein, and drug interactions with their target molecules is necessary. Furthermore, advanced tools enabling simulations of nanocarrier behavior in the bloodstream, in dependence of particle composition, size, charge and aspect ratio, as well as differing shear stress magnitudes and durations, are necessary to improve predictability of drug carrier behavior in the circulation.

## 2 Microscale and mesoscale modelling

Significant developments of computational modeling methods are rapidly bridging the gap between atomistic levels of description of both organic and inorganic molecular systems and multi-supra-molecular structures at the nanoscale, such as those including nanoparticles and other nano-engineered nanomaterials (ENMs) including nanocarriers (Bashiri et al., 2023; Cheng et al., 2023). At atomic levels of description, methods as molecular dynamics (MD) and mixed quantum-classical calculations, such as using quantum mechanical-molecular mechanics (QMMM) methods have seen tremendous developments and recognized with the 2013 Nobel Prize in Chemistry awarded to Karplus, Levitt and Warshel (NobelPrize, 2013). As stated by the associated press release, computational modeling become “.your Virgil in the world of atoms” as “Today the computer is just as important a tool for chemists as the test tube. Simulations are so realistic that they predict the outcome of traditional experiments.” In the same release, it is also notably highlighted the key application of computational modeling to performing “simulations of how a drug couples to its target protein in the body” though the challenge of attaining both accuracy and atomistic detail of physico-chemical description remains (NobelPrize, 2013). Thus, the last decade has seen rapid advances in computational modeling studies aimed at bridging the gap between the more accurate but computationally expensive atomistic and molecular-level simulation methods and the mesoscopic, nano-level coarse-grained modeling methods that are capable of including, within good accuracy limits, both biomolecular and inorganic (i.e., ENMs) systems (Mahmoudi, 2022; Bashiri et al., 2023).

**FIGURE 2:**
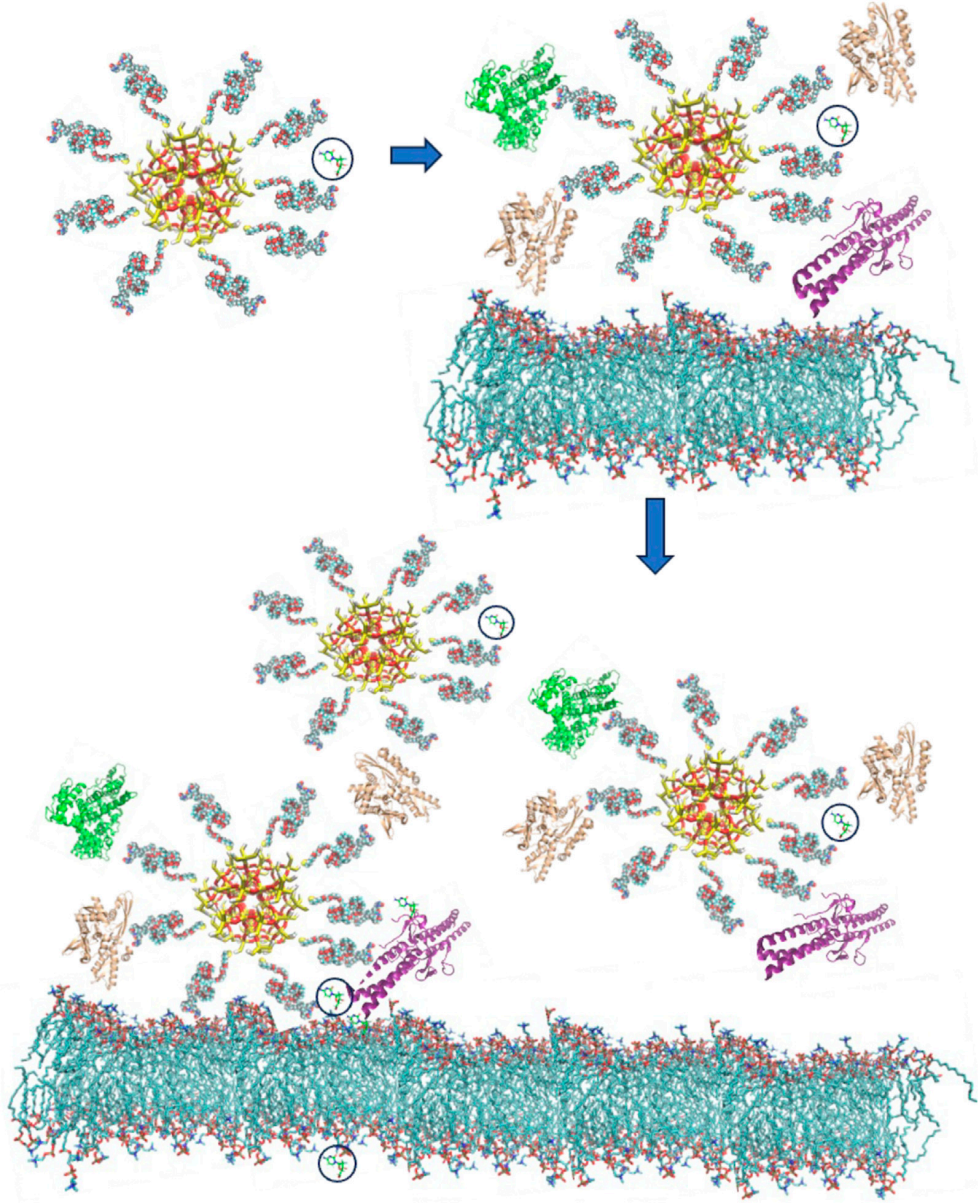
Schematic representation of the three main conceptual stages of interaction between a functionalized NC particle carrying a drug molecule (highlighted with a black circle) as it approaches a biological cell. Upon entering the biological environment (e.g., insertion in a blood vessel in the case for IVDD), the NC-drug system recruits additional biomolecules, especially proteins (e.g., shown in a cartoon representation as green, brown, or purple). In the final stage (bottom) the corona-covered NC interacts with the cellular membrane and, possibly with other NCs, and the complex process of drug release is initiated.

The current developments in the computational modelling for IVDD using nanocarriers (NCs) can be summarized in three major conceptual stages based on their associated interaction processes. For simplicity, here we will focus on NPs as the main NCs, though the same physico-chemical considerations can be applied in the case of other NEM systems (Rizvi and Saleh, 2018; Nasra et al., 2022; Yang et al., 2022). These three conceptual stages, illustrated in Figure 2, mirror the chemical processes occurring during the NP’s exposure to the biological environment:

(i) NP’s functionalization and drug loading,
(ii) formation and dynamic alteration of nanoparticle protein corona (NPPC) structures on the NP in the bloodstream, and
(iii) interactions of NPs with cell membrane and drug release.

### 2.1 Functionalization of NPs and drug loading

The effects of NP functionalization, grafting density and role of different PEG conformations in modulating the function of the liposome was simulated using coarse grained molecular dynamics (CGMD) techniques (Lemaalem et al., 2020). CGMD can capture the behavior of liposomes made using PEG-coated dipalmitoylphosphatidylcholine (DPPC) membrane bilayers and using MARTINI (Lee et al., 2009) beads for CGMD modeling in an aqueous environment (Lemaalem et al., 2020). The mean squared displacement can be monitored for observables such as the membrane changes during transition of the molecular spacers from mushroom-like into brush-like, more elongated conformations. This particular functionalization can be important in enabling PEG coatings to maintain lubricative property of the liposome and thus can be useful in designing cargo systems for drug delivery (Lemaalem et al., 2020).

**FIGURE 3:**
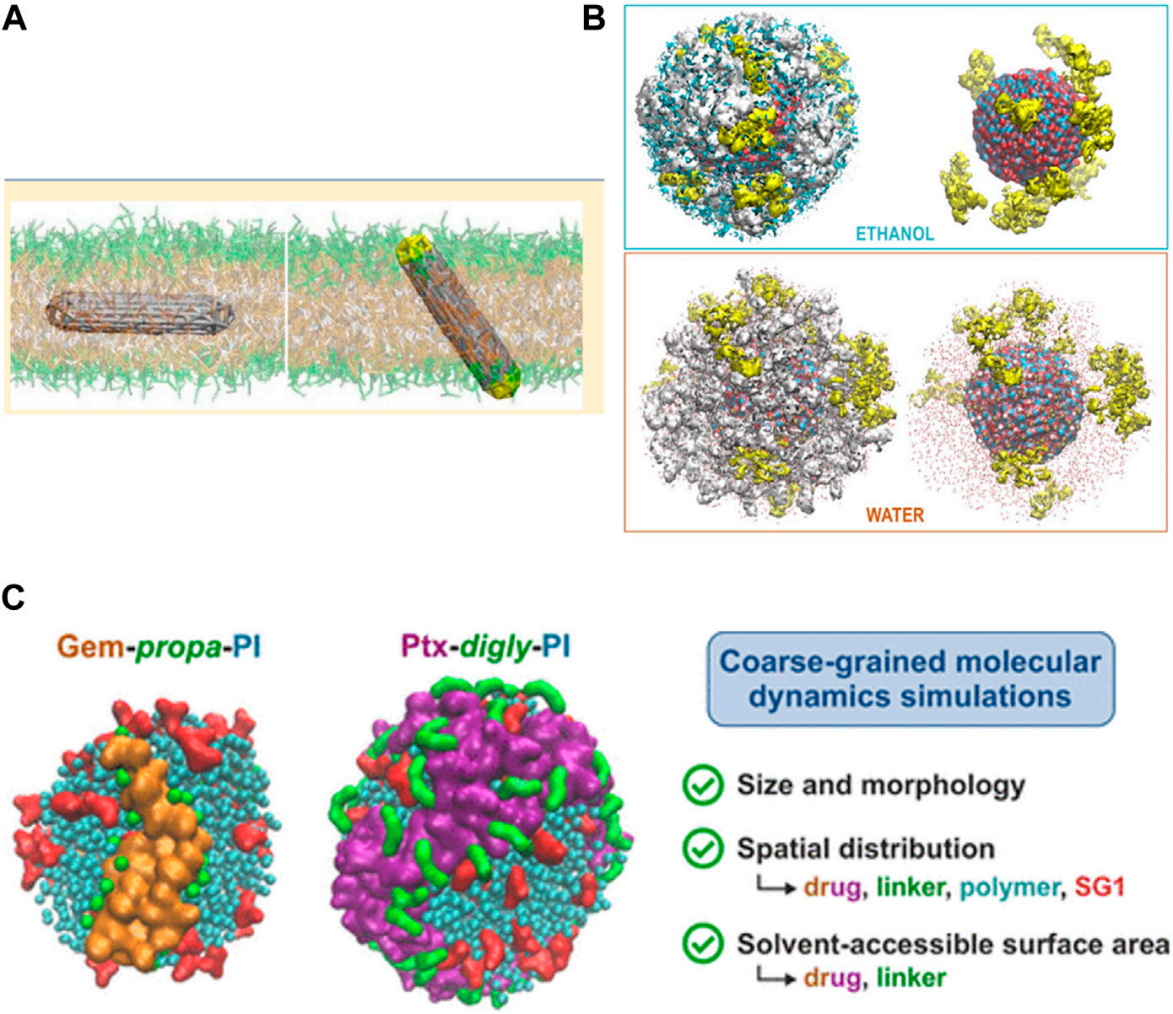
Illustration of three different NC systems modelled recently using MD: **(A)** Penetration of carbon nanotube NCs in bilayers. [Reprinted with permission from Baoukina et al. (2013)]. Copyright 2013 American Chemical Society). **(B)** Different density distributions of the drug CZX (yellow) in different solvents for a ZnO NP; Oleic acid spacers are shown in while, Zn in black and O atoms in red. [Reproduced from Trouki et al. (2022)], with permission from the Royal Society of Chemistry). **(C)** Different polymer prodrug NP system combinations: using different drugs (gemcitabine or paclitaxel anticancer drugs), linkers (e.g., propanoate or diglycolate), polymers (e.g., polyisoprene), and a capping agent (SG1). [Reprinted with permission from Gao et al. (2021). Copyright 2021 American Chemical Society).

Additional examples (Figure 3) which are addressable via all-atom MD/CGMD simulations to probe the choice of carbon nanotubes (CNTs) as possible IVDD vehicle have also been proposed (Mollazadeh et al., 2021). Tunning amino acids type, sequences, and the stoichiometry of CNT-attached self-assembled peptides, are also shown to improve dispersion of CNT; whereas PPI (i.e., polypropylene imine) dendrimers and PAMAM with specific branching densities in vicinity of PEG, are found to form cumbersome architectures and thus block the release of the drug from CNTs (Kavyani et al., 2018). The binding affinity between anti-cancer drug penicillamine and CNT is increased in presence of carboxyl functionalization. Similarly, for oxorubicin drug delivery, N-isopropyl acrylamide (NIP) enhances thermal stability of CNT. Co-loading and release of two anti-cancer drugs in chitosan-modified CNTs is also analyzed using simulations (Karnati and Wang, 2018). Penetration of doxorubicin-CNT into the lipid bilayer is studied and observed using steered MD, and it is shown that parameters such as the suitable length of the CNTs employed and their preferred orientation in membrane are examples (Sahoo et al., 2018) of different important parameters that one can infer through MD simulation in the course of IVDD mechanism (Mollazadeh et al., 2021).

Umbrella sampling is another technique that can be used to compute quantities such as the potential of mean force (PMF) for releasing certain drug molecules in the cellular medium (Allen and Tildesley, 2017; Frenkel and Smit, 2023). In recent studies (Moradi et al., 2018; Alinejad et al., 2020), this method is applied to study graphene oxide (GO) modified by longer PEG chains or folic acid to enhance the availability of drugs in cell. The penetration of gold NPs (Zolghadr and Moosavi, 2019) and carbon dots (Erimban and Daschakraborty, 2020) from water to DPPC/phosphatidylcholine membrane was estimated quantitatively using free energy calculations. The self-assembly of Janus lipid dendrimers (Elizondo-García et al., 2018) or drug release for a pH sensitive lipid-dendrimer (Otto and de Villiers, 2018; Maji et al., 2019) was studied in terms of binding energy, center of mass, solvent accessible surface area (SASA), radius of gyrations or contact map fluctuations.

### 2.2 Nanoparticle protein corona structures in the bloodstream

Regarding the second stage of formation and evolution of NPPC structures, kinetic models (Dell’Orco et al., 2012; Darabi Sahneh et al., 2013; Dogra et al., 2019) in cancer nanomedicine were used to interpret association and dissociation of plasma proteins like human serum albumin (HSA), HDL over NP, it highlights the possible role of NPs size in corona formation. CG-MD simulations offer another popular approach to model corona formation processes that was also used to model NP-corona interactions (Tavanti and Menziani, 2015).

To make connection and compare with experiments, Lobaskin et al. used a Kinetic Monte Carlo approach (Rouse and Lobaskin, 2021) to simulate the formation and equilibrium of the NP protein corona composition for silica NPs, both with and without PEG coating (Hasenkopf et al., 2022). Using a coarse-grained representation and rigid modeling of proteins, very good correlations of the calculated and experimental quantities (e.g., adsorption energies for several proteins) are found (Subbotina et al., 2023).

Recently, motivated also by experimental efforts to quantify the important role of the surface hydrophobicity for NP coronas (Mahmoudi, 2022), Buchete et al. developed a multiscale approach based on a stochastic method for protein docking of proteins on small silica and titania nanoparticles (Annapoorani et al., 2023; Mancardi et al., 2023). Due to computational constraints current implementations are limited to modeling only relatively NPs with relatively small diameters (i.e., 2–5 nm) covered by only a few tens of proteins However, the resulting models of NPPC structures have atomistic resolution and allow the efficient calculation of atomistic descriptors such as the hydrophobic or charge fractions of the solvent accessible surface area, with their corresponding statistical errors. These descriptors are important for the following stage of assessing the processes and interactions of protein-covered nanomaterials with different types of cells, tissues, or other biomolecular targets (Annapoorani et al., 2023; Mancardi et al., 2023).

### 2.3 Interactions of NPs with cell membrane and drug release

Regarding the third stage of interactions between NCs with target tissues and cells, the NPs size, shape and nature of functionalization may also modulate transportation barriers, penetration depth and immune responses faced by NPs within biological tissues (Islam et al., 2017). In this context, a multiscale Brownian dynamics-based model is proposed (Islam et al., 2017) to study dynamics of NPs in an artificial cell-free system as well as in a heterogeneous porous medium. Here, stationary circles of radius 10 μ are distributed randomly within a 40% region of a rectangular box to mimic porous capillary walls of the tissue, whereas NPs are considered as mobile spheres of different diameters with no mutual interaction (Islam et al., 2017). Velocity, interaction potential between NPs and cell tissues, diffusion coefficients of NPs after entering the box are computed over the simulation using the method of regularized stokeslets (MRS), i.e., a Lagrangian approximation of the Stokes equation. Similarly, interaction between NPs and cells are generated mathematically as probabilities for the NC of either getting captured in the cell wall or being bounced back in cellular fluid. However, this model ignores the electrostatic effects originated by NPPC structures in the bloodstream. Finally, the model reveals that, in physiological conditions, the sizes of the NPs has relatively small effects in membrane penetration, although it may play pivotal role when designing IVDD systems using artificial membranes.

In a different study (Tavanti and Menziani, 2015), a lumen structure with evenly implemented stents was used to model the arterial wall coated with PLGA. The length, density of polymers and the curvature of artery walls are modelled in simulation. The loss of PLGA mass over the simulation, affected drug diffusion pattern which implies gradual degradation of PLGA and release of the drug in artery; the drug binding was further estimated via its distribution profile over artery wall. Nonetheless, this model has limitations as it excludes diverse physiological conditions.

With regard to the efficiency of the drug release stage, the cleavage of the drug-polymer linker of a prodrug conjugate, under specific cellular conditions (e.g., lower pH in cancer tumours) is an important parameter that needs to be studied and understood in detail. A CGMD study (Gao et al., 2021) on different prodrugs consisting diverse drug-linker-polymer combinations (either a gemcitabine or a paclitaxel anticancer drug, either a propanoate or a diglycolate linker, and a polymer named polyisoprene, Figure 3) was performed; supramolecular assembly, solvation free energy of such systems is calculated over simulated trajectories. It was observed that gemcitabine becomes more exposed, but paclitaxel remains buried, similarly the bioavailability for all the drug-polymer linkers shows differences as per observations. This justifies usage of MD to model suitable cargo for drug release in targeted area.

The interaction between two anticancer drugs named flutamide (hydrophobic) and glutathione (GSH, hydrophilic) with a model membrane composed of DPPC lipids, in their free as well as in gold NP loaded states, was also studied using MD (Farhadian et al., 2022). Here, gold nanoparticles enhanced permeability of pure GSH within DPPC, whereas for hydrophobic flutamide the diffusivity is decreased. However, the formation of inactive complex by flutamide over bilayer is also an important step, as it blocks androgen receptor. Thus, MD can also reveal different drug release mechanisms and their impacts to achieve desired goal.

The role of pH in a diseased cell was further investigated for two different dendrimers named PPI and PAMAM at three different pH of solutions. The simulations (Gupta and Biswas, 2018) focused on changes of fractal dimensions as well as changes in conformational fluctuations of those dendrimers as function of pH. Thus, one can design the most suitable one after gaining insights from simulated data (Gupta and Biswas, 2018).

Additionally, NPs can also lead to cytotoxicity in vicinity of membrane, as it may alter shape, curvature, thickness or order parameter of hydrophilic tails leading to leakage or pore formation within bilayer (Song et al., 2012; Ding and Ma, 2018; Bunker and Rog, 2020); thus, a balance between increased therapeutic efficiency and decreased cytotoxicity of NCs is demanded. Specifically, MD simulations (Dallavalle et al., 2015; Mhashal and Roy, 2016) reveal that smaller size of gold or graphene NPs has minimalistic effects on lipids fluidity, whereas the larger size show prominent effects. On the other hand, a CGMD study (Lin and Gu, 2014) indicates that surface roughness and spacer densities of NCs can also affect phase transition temperature of bilayer. Shi et al. (2011) demonstrates that NCs with small cap can orient themselves in a vertical manner within bilayer but the elongated nanotube/nanosheets with sharp edges require larger binding energy, which gradually leads to membrane rupture.

Additional crucial parameters are ligand distributions, orientations, or the ligand arrangement pattern over NC’s surface for specific receptor binding within cell, their potential role was mentioned in MD and Monte Carlo simulations (Martinez-Veracoechea and Frenkel, 2011; Liu et al., 2016; Angioletti-Uberti, 2017). Thus, one needs to consider all these aspects while designing NCs for IVDD.

A system of zinc oxide (ZnO), decorated with oleic acid chains and loaded with the drug carfilzomib (Figure 3) was simulated using ReaxFF MD (Trouki et al., 2022). Here, the self-assembly pattern of NC, the binding between drug-NC, the dynamics of the entire cargo, release of the drug within solvent was analyzed theoretically.

To sum up, enabled by the impressive growth of computational atomistic and molecular modeling methods developed in recent years, the field of computational modeling of IVDD using NCs is reaching new frontiers. This growth was accelerated by advances in the availability of (i) faster and affordable high-performance computer (HPC) hardware resources, (ii) multiscale, both atomistic but especially coarse-grained-based modeling approaches, and (iii) revolutionary parameterization methods steaming from machine learning approaches that allow simplified yet accurate modeling and simulation methods to overcome their accuracy and efficiency-related limitations.

## 3 Macroscale modelling

The convection and diffusion properties of nanoparticles in flow media and particularly in blood affects their ability to interact with the vascular wall. Experiments show that there is a considerable effect of particle size along with hemodynamics, blood rheology, and vessel size on the adhesion efficiency (Charoenphol et al., 2010). However, experimental setups can be costly and difficult to construct. Further to their ability to simulate atherosclerosis formation (Avgerinos and Neofytou, 2019), macroscale modelling provides a cost-effective alternative method for this kind of tests and can assess the effects of parameters, including magnetic field strength (MFS), magnet size, particle size, the initial position of particles, and the relative magnetic permeability of particles, on the efficacy of MDT through the artery walls are characterized (Manshadi et al., 2018; Mahmoodpour et al., 2020). In addition, the optimal particle size range for which the surface density of particles (SDP) adhered on the plaque lumen reaches its maximum can be specified (Shamloo and Forouzandehmehr, 2019). Optimization of physical parameters of such particles and the magnetic configuration, estimating the fraction of particles reaching a given target site in a large patient-specific vascular system can also consider different physiological states (heart rate, cardiac output, *etc.*) (Patronis et al., 2018). Macroscale modelling *in silico* methods can also study different classes of magnetic cores and the thickness of biocompatible coating materials (Lunnoo and Puangmali, 2015). Novel computer simulation models have been developed to study nanoparticle transport in a representative artery system, subject to shear-induced diffusion of nanoparticles due to hydrodynamic interactions with red blood cells using computational fluid dynamics (Xu and Kleinstreuer, 2018) or Lattice Boltzmann methods (Liu et al., 2018). Patient specific geometries reconstructed from DICOM images, such of the coeliac trunk, can also be taken into account (Boghi et al., 2017). Particle agglomeration can also be considered to assess the NP bio-related capabilities.

### 3.1 Nanoparticles in bloodstream

The most widely used methods in simulating nanoparticles in fluid flow are *Lagrangian* and the *Eulerian* methods. The Lagrangian methods describe the path of each individual particle whereas the Eulerian methods simulate the particles as a whole, i.e., as a concentration with similar properties at certain different location of the domain. A further distinction concerns the numerical methods for simulation fluid flow namely, conventional *Computational Fluid Dynamics* (CFD) that solve numerically the Navier-Stokes equations that take into account macroscopically the pressure and the velocities of the medium and the *Lattice-Boltzmann Method* (LBM) where a state represents the position and the velocity of interacting pseudo-molecular particles of a fluid (Korolija et al., 2017). In addition to this, and in terms of the geometry used in the simulations, modeling approaches use either a *simplified geometry* which usually represents an axisymmetric model of an artery with a stenosis or an aneurysm (Neofytou and Tsangaris, 2006) or a *case-specific geometry* of an arterial segment that is derived from CT or MRI scans of a patient (Skiadopoulos et al., 2017). Furthermore, and in terms of the blood properties, blood can be simulated as a *Newtonian* or *Non-Newtonian* fluid. For the latter case, Non-Newtonian blood models are to be employed in the computations (Neofytou, 2004).

#### 3.1.1 Lagrangian methods

Roa-Barrantes and Rodriguez-Patarroyo (Roa-Barrantes and Patarroyo, 2022) studied the kinetic behavior of a magnetic nanoparticle subjected to external magnetic fields in an arteriole axisymmetric model with pulsatile flow. They gave the trajectories of the particle with dependence on its starting position and magnetic intensity. A tube model of an artery was also used by Lee et al. (2022) to conduct a parameter study to show the effects of changing the dipole moment, the distance from the magnet to the blood vessel, and the initial release point of the nanoparticles using the Carreau model for modelling the rheology of the blood. A simplified geometry was used by Hussain et al. (2023) depicting an intracranial aneurysm where the effects of Ag nanoparticles are studied. A 2D bifurcation was employed by Larimi et al. (2014) to simulate the flow and magnetic targeting of SPIONs as novel drug delivery vehicles with particle tracking to estimate particle behavior under influence of imposed magnetic field gradients along the bifurcation.

Qiao et al. (2022) used idealized three-dimensional geometric models of coarctation of the aorta for their simulations and the goal was to propose a method to calculate the shear stress thresholds for the diseased aorta. In this respect and although their work does not include nanoparticle dispersion calculations, it preliminary demonstrates the feasibility of shear-activated targeted nanoparticle drug delivery in the treatment of aortic diseases. Tripathi et al. (2021) studied the unsteady hybrid-nanoparticle (silver and copper) hemodynamics in an axisymmetric model of an inclined artery with mild stenosis and aneurysm features. A Finite Difference Scheme together with the Carreau fluid model whereas the NPS were considered to have various shapes (bricks, cylinders, platelets, blades). Results showed that the inclusion of hybrid nanoparticles (Ag–Cu/Blood) within blood increases the axial velocity and temperature magnitudes more significantly as compared to unitary nanoparticles (Ag/blood) at both the stenosis and aneurysm segments.

As opposed to simplified geometries, Shamloo et al. (2019) numerically reproduced the arterial geometry by image processing of CT-scan images of a case specific coronary arterial segment. They implemented the finite element method together with Fluid-Structure Interaction (FSI) capabilities simulation based on physiological boundary conditions and using the non-Newtonian Carreau-Yasuda model for the blood. For the particle dispersion, a Lagrangian description of Magnetite particle dynamics considering momentum equation of motion for each particle under an imposed external magnetic field was assumed. A patient-specific geometry was also used by Boghi et al. (2017) and was reconstructed from a data set of CT scan images of the coeliac trunk of a middle-aged healthy man. Nanoparticles, used for drug targeting for the treatment of hepatic cancer, are supposed to be dragged into the liver by an external magnetic field which was also taken numerically into account by the Maxwell equations. For the simulations, the CFD package OpenFOAM was used that couples the Lagrangian particle dynamics with the Navier–Stokes equations. A widely used CFD package namely, ANSYS Fluent™ was used by Hewlin et al. (2019) for the fluid-particle flow simulation in a patient-specific domain of a diseased left carotid artery bifurcation. The model is used to investigate the transport and targeting efficiency of magnetite micron range particles taking into account an external magnetic field as a way towards effective treatment of both cancer and cardiovascular disease. The latter disease is also the focus of Suo et al. (2012) who simulated the flow in a patient specific part of the left coronary artery to examine the feasibility for treatment with iron nanoparticles and by modeling hypothetical magnet fields. Lindemann et al. (2021) used the finite element method (FEM) of SPIONs in a capillary sized vessel network to study the effect of the magnetic field gradient as well as of magnet distance to the vessel geometry, magnetic flux density of the magnets, SPIONs hydrodynamic diameter and magnetic moment on the Magnetic-drug-targeting effectivity. Aryan et al. (2022) also used FEM aiming at enhancing the SPION delivery to the desired branch of a 3D patient-specific carotid bifurcation using four different magnet configurations implanted adjacent to the artery wall. They employed the non-Newtonian Carreau model for the blood flow and the results show that it is possible to guide more particles to the desired branch (up to 4%) while preventing particles from entering the unwanted branch (up to 13%). A numerical study into nanoparticle transport in a bifurcated carotid artery, and a scaled-down version approximating an arteriole and branch capillaries, has also been conducted by Doig et al. (2012) using an in-house code to establish the influence of size on the trajectory and residence time of particles.

With respect to LBM, Patronis et al. (2018) developed a model for the simulation of magnetic particles in a patient-specific circle of Willis, the central blood distribution system in the brain, in order to determine the fraction of injected particles that reach a defined target site under varying physical parameters (of the nanoparticles) and physiological states (of the patient) using an external stationary magnet. The LBM can be combined with a spectrin-link method for RBCs, and a Langevin dynamics (LD) approach to capturing the motion of the suspended NPs to fully resolve the NP transport in cellular blood flow (Liu et al., 2018). The deformability of particles can also be taken into account by employing a Moving Least Squares (MLS) approach to accurately interpolate the pressure, velocity and force fields between the Eulerian and Lagrangian meshes describing the fluid and the capsule dynamics, respectively (Coclite et al., 2019) thereby addressing particle flows in capillaries. Tan et al. (2016) carried out a numerical study on NP transport and dispersion in red blood cell (RBC) suspensions under shear and channel flow conditions, utilizing an immersed boundary fluid-structure interaction model, an elastic cell membrane model and a particle motion model driven by both hydrodynamic loading and Brownian dynamics. This model accounts also for RBC deformation and results confirm previous findings that the NP dispersion rate is strongly influenced by local disturbances in the flow due to RBC motion and deformation.

#### 3.1.2 Eulerian methods

The treatment of hepatic cancer was also the scope of Xu and Kleinstreuer (2018) that used a geometry representative of the human hepatic artery system to study nanoparticle transport subject to shear-induced diffusion of nanoparticles due to hydrodynamic interactions with red blood cells. The blood flow was described non-Newtonian model capturing the shear-thinning behavior at elevated shear rates whereas the convection-diffusion equation was employed to describe nanoparticle transport, incorporating both Brownian as well as shear-induced diffusion effects. A 2D collapsible tube was employed by Aminfar et al. (2013) to investigate the laminar flow of a ferrofluid consisting of blood and Fe_3_O_4_ spherical shape nanoparticles under the influence of different magnetic fields. A 3D Y-branched artery-model that is supposed to supply a tumor region was considered by Gitter and Odenbach (2013) who carried out FEM of the flow simulating the blood as a Carreau -Yasuda fluid to account for its shear-thinning properties. The purpose was to study drug-loaded nanoparticles concentrated within a target region due to the influence of a magnetic field. Poonam et al. (2022) investigated the consequences of nanoparticle addition on blood flow passing through a curved artery model with mild stenosis and aneurysm conditions. They used the Crank-Nicolson method is applied to solve governing equations and showed that with increase in volume fraction of gold (Au) nanoparticles, the velocity profile rises, while, reverse effect is noticed for the volume fraction of aluminium oxide (Al_2_O_3_) nanoparticles. The inclusion of inertia in NP dispersion in biological flows was taken into account by Pilou et al. (2011) to study the dispersion and impingement on the wall of particles of various sizes in a bend and subsequently in a physiologically realistic bifurcation (Pilou et al., 2013).

### 3.2 Nanoparticle agglomeration

NP agglomeration strongly affects their biodistribution, thus determining reactivity, toxicity, fate, transport, and risks. NP agglomeration is induced by the collision frequency and the collision efficiency of the phenomenon. The collision frequency is the mechanism that brings NPs in contact and is governed by Brownian diffusion, fluid motion and differential sedimentation. The collision efficiency is the mechanism that makes NP stick together and is based around the Derjaguin-Landau-Verwey-Overbreak (DLVO) or the extended DLVO (xDLVO) theory (Hotze et al., 2010), according to which the sum of attractive and repulsive forces between NPs such as Van der Waals, Electrostatic and Magnetic forces determines the intensity of agglomeration. Numerical modelling of aggregation/agglomeration is given by the discretised form of the Population Balance Equation (PBE) (von Smoluchowski, 1917) and predicts the evolution of the aggregates size distribution and includes parameters to account for the kinetics and the dynamics of the phenomenon.

Exploiting this, Atmuri et al. (2013) performed integrated experimental and modeling study to explore aggregation in concentrated attractive colloidal suspensions with the use of the PBE model that includes the full calculation of the stability ratio as well as variations in DLVO interactions between particles and aggregates as aggregates grow. The model successfully predicts aggregation regimes of charged colloidal particles over a range of salt concentrations and particle concentrations. Runkana et al. (2004) attempted to integrate the theories of surface forces in presence of polymers with the population balance framework to model polymer-induced flocculation. They solved the geometrically sectioned population balance equation (PBE) according to the scheme by Hounslow et al. 1988) where collision efficiency was estimated as a function of van der Waals attraction, electrical double layer repulsion and bridging attraction or steric repulsion. Biggs and Lant (2002) also applied the method developed by Hounslow et al. (1988) for modelling activated sludge flocculation, providing a good approximation of the change in mean floc size with time.

Liang et al. (2016) used a different kind of sectioning of the PBM for aggregation of particles as, namely, the one proposed by Kumar and Ramkrishna (1996) to predict aggregation behavior of submicron-sized particles of praseodymium-doped zirconium silicate in aqueous suspension. The model was also based on the xDLVO theory and was further modified to predict the stable state of the aggregation by introducing the volume mean size of the aggregate to the stability ratio. The same method of sectioning of the PBM was used by Huang et al. (2020), who studied the dispersion/aggregation behavior of colloidal particles in the suspension under different conditions. The colloidal system consisted of a dispersion of praseodymium-doped zirconium silicate pigment in an aqueous suspension by modified hydroxyl copolymer where the interactions between the models were modelled based on the xDLVO theory and an experimental study was also performed, with the authors reporting that predicted data are similar to the experimental results.

Grabinski et al. (2016) studied the implications of agglomeration and unique transport kinetics of NPs in biological media affecting cellular dose in typical *in vitro* toxicity experiments involving exposure of a mono- or co-culture of cells to nanoparticles (NPs). For this a transport model was used to approximate the shear rate experienced by the cells in dynamic conditions to evaluate physiological relevance. The transport kinetics show that NP behavior is governed by both gravity and diffusion forces in static conditions and only diffusion in dynamic conditions. Neofytou et al. (2022) performed simulations solving the PBE in conjunction with the xDLVO theory using the Kumar and Ramkrishna (1996) method for assessing the colloidal stability and aggregation tendency of iron oxide nanoflowers (IONfs) in biofluids. Colloidal stability experiments were also conducted on IONfs in aqueous phosphate buffered saline solution. The simulations showed a slight overprediction of the aggregation rate compared to the experiments and this could be attributed either to the patchiness on the NP surface, or to the fractal nature of aggregates that could not be considered in the model.

### 3.3 Nanoparticle targeting

Simulations of nanoparticle targeting aim at (i) assessing the effect of an externally applied magnetic field on flow of NPs in the circulatory system and ultimate reaching their target-tissue and (ii) looking into the NP’s ability to adhere on the target-tissue depending on the molecular functionalization of NPs intended for binding to a specific receptor in the macroscale by applying proper boundary conditions.

#### 3.3.1 Magnetic targeting

**FIGURE 4:**
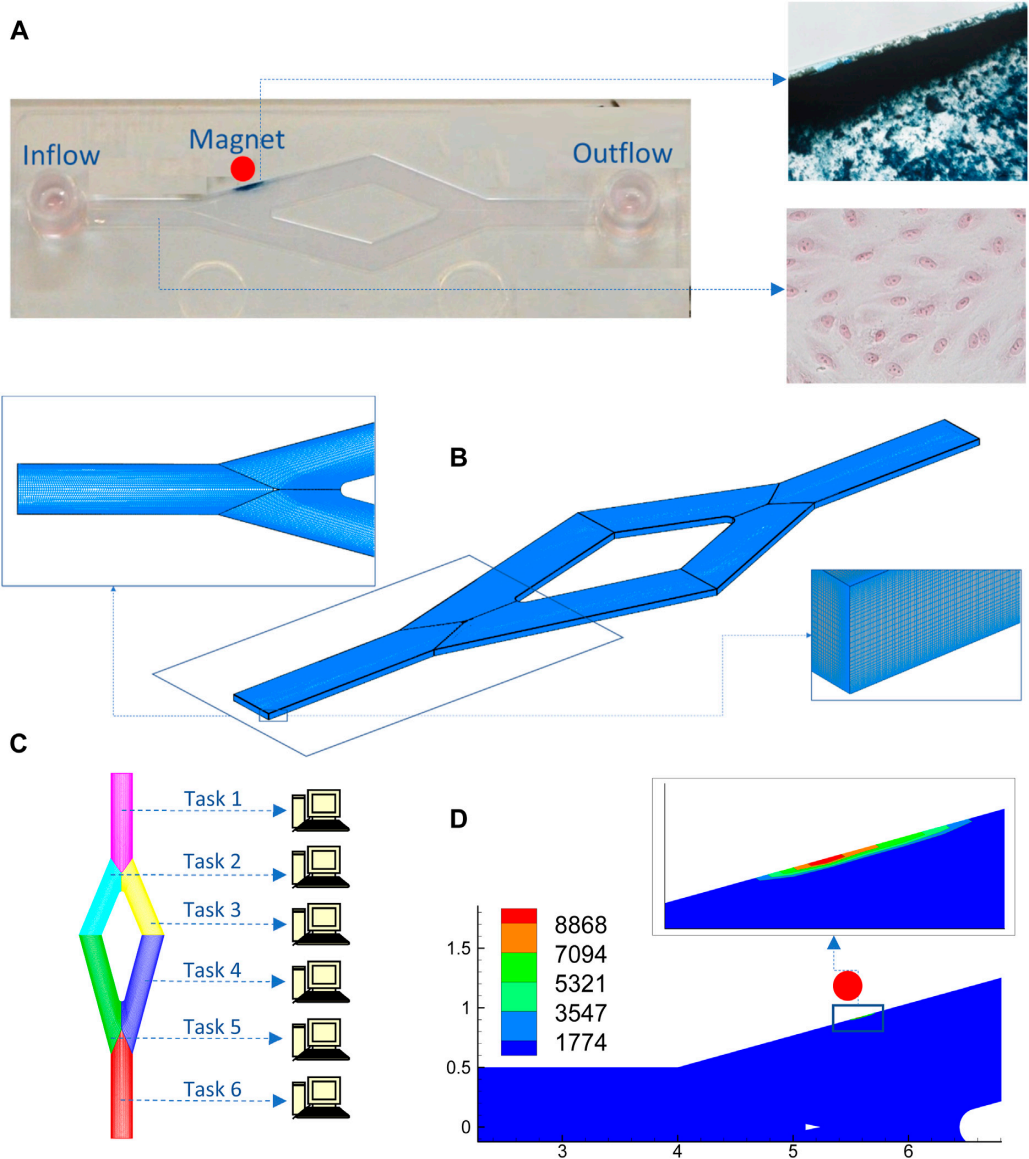
Macroscale simulation of an *in vitro* experiment of a SPIONs solution that is circulating through a channel and is under the influence of an external magnetic field: **(A)** *In vitro* slide image (left) with a monolayer of endothelial cells at the bottom (lower-right panel) and accumulated particles (right upper-panel); **(B)** Grid for flow-particle dynamics simulation with boundary fitting topology (left) and refinement applied near the wall (right) to better capture localized effects; **(C)** Grid with a multiblock architecture enabling parallel processing; **(D)** Simulation and results regarding particle internalization using a code encompassing a module for particle dispersion and agglomeration, a module for external magnetic fields and particle-depended wall boundary conditions.

Magnetic targeting simulation (Figure 4) provides insight into the attributes of the magnetic field that is needed to achieve the desired NP-accumulation outcome avoiding thereby the necessity of experimental setups that would have been otherwise costly especially when a parametric study is required regarding magnetic efficiency based on magnet positioning and also both geometric and magnetization attributes of the NPs. Modelling usually facilitates the analytical expressions of the magnetic field induced by a stationary magnet such as bar magnet, circular loop, infinite wire etc (Smythe, 1950).

A range of magnetic field strengths were modeled by Lee et al. (2022) for the motion of superparamagnetic nanoparticles in a tube model using equations for both a bar magnet and a point dipole and it was shown that the bar magnet is effective at capturing nanoparticles in limited cases, while the point dipole is highly effective across a range of conditions. The same geometrical setup and the magnetic field by a cylindrical external permanent magnet was used to specify trajectories and capturing of magnetic nanoparticles coated with drug agent injected at the entrance of a vessel and captured by a magnet located at a specified location where tumor exists (Sharma et al., 2015; Shazri and Idres, 2017). A tube model was also used by Roa-Barrantes and Patarroyo (2022) with a magnetic field produced by an infinite cylindrical magnet to study the trajectory of a magnetic NP in pulsatile flow. Manshadi et al. (2018) used a four-layer structural 2D model of the artery tissue and the magnetic field around a rectangular prism to study the delivery of both clusters of drug micro- and nanoparticles and single drug/cargo into the luminal artery for curing inflammation, and their translocation through the artery layers. A 2D channel model of a vessel was also used by Mahmoodpour et al. (2020) together with a cylindrical permanent magnet positioned below with varying-magnetic-field-intensity options to study two cases of initially uniform distribution of drug carrying particles in the blood flow and separate injection of the particles upstream of the target area and showed that capture efficiency increases with increase of the magnetic field intensity and particles diameter and decrease with the blood flow velocity. The magnetic field created by a solenoid situated near a 2D channel model with a stenosis was used to show that it provokes the accumulation of magnetic NPs in stagnant zones with vortices induced in the flow field and appear near the stenosis (Starodumov et al., 2023). A more complex 2D arterial model with multiple bifurcations together with a permanent magnet positioned at its side is used to computationally study the size-dependent capture efficiency of Fe_3_O_4_, Fe_2_O_3_ and Fe NPs in mimicked arterial flow (Lunnoo and Puangmali, 2015). Carrier sizes of 10 nm-4 μm in radius were considered and results show that particles larger than 2 μm were efficiently captured at the desired location by the external magnetic field, and the capture efficiency was approximately 95%.

A *case specific* Left Anterior Descending (LAD) coronary artery geometry was used by Shamloo et al. (2019) together with an externally imposed magnetic field with a specific designated direction that would significantly elicit particles to move toward the vessel wall (margination). Under the influence of the magnetic field, the optimal particle size scope for which the surface density of particles adhered on the plaque lumen reaches its maximum was numerically specified. In another case-specific study the magnetic field of a cylindrical electromagnet was used to assess particle capture efficiency in a diseased left carotid bifurcation artery using the magnetic properties of magnetite and equations describing the magnetic forces acting on particles produced by an external cylindrical electromagnetic coil and achieving capture-efficiency up to approximately 98% (Hewlin et al., 2019). A 3D geometry representation of a bifurcating *in vitro* setup was used by Tsimpoukis et al. (2023) together with a permanent magnet placed at the bifurcation and modelled as an infinite wire to assess the localized accumulation of particles with and without magnetic field.

A *combination* of stationary magnets namely, twelve small permanent magnets positioned in three consecutive quadrupole arrangements were placed around a tube model of an artery and the flow-particle field was computed so as to predict the amount and position of particle complexes which can be retained by the imposed magnetic field (Heidsieck et al., 2012). The purpose was to distribute complexes consisting of lentivirus and magnetic nanoparticles as homogeneously as possible along a perfused murine aorta as a part of regenerative medicine in the cardiovascular system aiming at the restoration of the healthy physiological conditions to the diseased heart and the vascular system. Lindemann et al. (2021) carried out simulations of Magnetic drug targeting tracing single SPIONs in a 3D geometry of eight multibranched vessels with sizes in the range of capillaries assuming two different configurations of magnet arrays, i.e., 6 bar config-uration and a 5 bar Halbach configuration. The results show that the magnetic targeting effectivity is up to 32% higher than the one calculated without the presence of a magnetic field.

#### 3.3.2 Adhesion on tissues

Adhesion is a physical mechanism that depends on a variety of parameters that range from the local fluid (blood) flow conditions, especially shear rate, to the size, shape and ligand surface decoration of NPs and the availability of the corresponding receptors on the vasculature wall at the site of interest. The simulation of this mechanism is usually defined by a boundary condition that is imposed when the fluid-particle equations are numerically solved.

The effort towards simulation adhesion mainly stems from the need to assess by modelling the deposition of particles suspended in fluids that flow over adhesive walls which are facilitated by the formation and dissociation of biological bonds between receptors and ligands. Adhesion of microparticles such as monocytes and platelets to a vascular surface are usually modelled by a boundary condition for a reactive surface equating mass diffusion at the wall with the adhesive flux (David et al., 2001; Longest and Kleinstreuer, 2003; Weller, 2008; Tokarev et al., 2011). In other words, the adhesive flux given as the product of the particle concentration at the wall times the adhesion rate constant (which depends on parameters such as the binding strength, shape, size, local shear stress, etc.) equals the flux of the particles arriving at the wall by diffusion that is given as the product of the partial derivative of the concentration near the wall to the distance from it times the diffusion coefficient. This equation can be extended to include a term for the dissociation of bound particles (Lyczkowski et al., 2009; Hansen et al., 2012) as a product of the concentration of bound particles times a dissociation constant depending on parameters similar to the adhesion rate constant.

This equation was tailored for predicting delivery carriers across the nano-metric length scale using 43 nm and 1.1 mm diameter polystyrene spheres (Haun et al., 2010) where monoclonal antibody specific for ICAM-1 is utilized as the targeting receptor and adhesion to ICAM-1 coated glass substrates in a parallel-plate flow chamber at physiological shear rates is quantified. This equation was further used to elucidate the effects of particle size on the selective binding efficiency of nanoparticles to vessel wall that experiences temporal and spatial variations in wall shear stress and where non-Newtonian viscosity characteristic of blood was incorporated by using the Carreau model (Kim and Rhee, 2011; Jeong et al., 2013).

The adhesion rate constant for microparticles (MPs) can be given as a function of a probabilistic adhesion model which gives the probability to create a biological bond when ligands on the surface of MPs are close to receptors on the vessel wall (Ye H. L. et al., 2019). Decuzzi and Ferrari (2006) developed a probabilistic model for the specific adhesive interaction between a non-spherical particle and a cell layer under a linear shear flow showing that the strength of adhesion is affected by (i) the *geometric features* of the particle, (ii) the *biophysical parameters*, such as the equilibrium separation distance between the substrate and the particle; the maximum distance at which ligand–receptor bonds can be formed; the ratio between the shear stress at the wall and the surface density of receptors; the surface density of the ligands and by (iii) the *biochemical parameters*, such as the characteristic length of the ligand–receptor bond and the characteristic affinity constant of the ligand–receptor pair. Hossain et al. (2014) exploited this function to predict the vascular deposition of blood-borne nanoparticles (NPs), loaded with therapeutic and imaging agents within an inflamed arterial tree. Results show the difference of adhesion attributes between NPs decorated with antibodies directed toward three endothelial adhesion molecules, namely, ICAM-1, VCAM-1 and E-selectin. Further study exploiting the same function (Hossain et al., 2013) shows that adhesion is dramatically affected by changes in patient specific attributes, such as branching angle and receptor density whereas the adhesion pattern correlates well with the spatial and temporal distribution of the wall shear rates. Pilou et al. (2021) used the same function to study particle adhesion on the diseased vascular wall of a patient-specific infrarenal abdominal aortic aneurysm with for a time-dependent flow field over a cardiac cycle. The effect of particle size for 50 and 500 nm nanoparticles and flow field on adhesion efficiency and location is investigated using CFD and it is found that 50 nm particles adhere diffusely on the vascular wall, whereas adhesion of 500 nm particles is highly localized. The same methodology was used to investigate the adhesion of superparamagnetic iron oxide NPs (SPIONs) under steady flow conditions in a bifurcating *in vitro* setup lined with primary human endothelial cells cultured at the bottom wall (Tsimpoukis et al., 2023). A similar probabilistic function was used by Ebrahimi et al. (2021) to evaluate the surface density of Nanocarriers adhered via ligand-receptor binding to the inner wall of an Abdominal aortic aneurysm (AAA) using CFD and FSI analysis for both patient-specific and ideal AAA models. Adhesion of particles over an atherosclerotic plaque located in the LAD artery of a patient was also considered by the same function (Shamloo et al., 2019). Coclite et al. (2017) present a modified probability of adhesion function based only on the forward bond rate, and on the number of ligands actually probing the surface, for predicting the adhesive interaction of particles with blood vessel walls under capillary flow using a combined Lattice-Boltzmann–Immersed-Boundary method.

## 4 Outlook

Nanocarriers developed for IVDD applications have shown clinical efficacy in improving bioavailability of drugs and extending the circulation half-life of encapsulated drugs or imaging agents. Based on the preclinical studies, they also have a great potential for disease targeting and improving the organ-specific accumulation. However, the loading/release rate of the loaded drugs and/or imaging agents, as well as the targeting features of nanocarriers are regulated by several and interconnected mass transfer and biochemical mechanisms occurring over multiple time and space scales (from nano to macro). These features depend on the nature of the nanocarrier, its properties like surface charge, size, hydrophilicity, *etc.*, as well as on the specific interactions between the drug and its carrier, not to mention the role of the solvent and of surface functionalization. Therefore, while a comprehensive understanding of these phenomena is far from being achieved by experiments alone, modern materials modelling techniques may accelerate the optimization of drug-loaded nanocarriers. This complexity can be managed by introducing *in silico* models to investigate the drug transportation, targeting and release mechanisms of multi-functional nanocarriers, with the final goal to significantly improve preclinical studies in terms of cost, time, and animal welfare especially in view of the growing public pressure against animal experimentation in most developed countries that is leading to the development of alternative methods for preclinical assessment.

*In silico* model development stems from the vision of replacing conventional (pre)clinical trials within silico methods and applies to the development of more efficient nanocarriers for intravascular therapies. The essence of the concept lies in breaking down the experimental stages of a preclinical trial for a novel nanoparticle and defining *in silico* tools that can model each stage and give credible results, enabling the establishment of a standardized method for *in silico* preclinical trials. The models developed, validated and exploited for the single stages of preclinical assessment of the nanocarriers should subsequently be integrated into a multiscale model suite.

Numerical simulations of the nanocarriers distribution in physiological and pathophysiological flow conditions, the effects of blood cells and hemodynamic forces on their margination in the vessels and the attachment to the endothelium before paracellular transport, can provide guidelines for optimized NC design. Similarly, modelling of the delivery of NCs to solid tumors can provide insights for the mechanisms of NC transport from the tumor vasculature into cancer cells. The development of *in silico* mechanistic models which will aim to accurately represent the multiple stages in preclinical trials such as biodistribution, toxicity and targetability studies addressed by MD, CGMD, Molecular Docking, PBPK and CFD models can lead to nanocarrier optimal-by-design modelling. This approach can combine novel and already available *in silico* tools that will be tailored on a case-by-case basis and will cover all stages from designing the nanocarrier to testing it in accordance with preclinical requirements. *In vivo*, *in vitro* and *ex vivo* data from literature or from design-by-case experiments should be stored on a sustainable database assisting to the development and exploitation of the *in silico* tools. This approach is expected to save costs and time required for the evaluation of the NCs. Given that preclinical studies are time-consuming, expensive and involve ethical issues that arise from the use of animals for experimental purposes, the establishment of specific guidelines for NC design is of great importance and priority.

Overall, atomistic MD and CGMD simulations can assist and have already been used in designing NCs for IVDD with suitable functionalization. Biophysical descriptors such as the density of spacer molecules, chain length, charge, or the type, shape, and conformations of NCs can be studied and tuned computationally. The binding affinity for the encapsulated drug can also be estimated using free energy calculations. One can study and predict the drug release dynamics (e.g., using umbrella sampling, steered MD, or Markov State Models). By including models of the cellular environment (e.g., the bilayer membrane), it is also possible to study the effects of the interaction between cells and the developed NC-based cargo to minimize toxicity. For larger systems, coarse grained MD and molecular docking can be used to accelerate studies that require longer simulation times. Computational methods for studying and quantifying the NC behavior at a molecular level are increasingly emerging as viable additional approaches to complement experiments for processes such as probing NC functionalization, drug loading, protein corona structures, drug release, or NC-membrane interactions to minimize toxicity and maximize efficacy in intravascular drug delivery.

Macroscale models such as CFD models can exploit the outcome of microscale model development in order to describe fluid particle interaction more accurately but also to define more accurate parameters for boundary-condition equations in terms of simulating large-scale particle-wall interaction that is triggered by receptor-ligand reaction at vascular areas of interest. In addition, microscale models can also assist towards developing more accurate macroscale particle-aggregation models that will better predict the colloidal stability of a system.

Based on simulation efficiency and high in vitro-in vivo extrapolation potential, a multiscale computational modeling framework for NC-based IVDD can greatly reduce the need for *in vivo* animal experiments especially in terms of determining plasma and organ tissue concentrations in, e.g., animal models for perfusion limited (or perfusion altered) tissue models such as tumors or necrotic tissue, first-pass metabolism, as well as absorption from the bloodstream. Successively, computational models are expected also to be able to support animal-to-human extrapolation. Furthermore, *in silico* modelling can in the future provide an automated and flexible protocol for the fast prediction of the drug release rate, response to the environment, and targeting of functionalized and drug-loaded nanocarriers. The integration of computational modeling methods in the regulatory process for nanocarrier development could drastically reduce the cost of innovation and, ultimately, the cost of healthcare. For this, further efforts are necessary to strengthen the reliability, efficacy and robustness of the *in silico* predictive multiscale models used for development and preclinical assessment of nanocarriers in IVDD considering comprehensively three key elements: physics-based theoretical accuracy, experimental validation, and uncertainty quantification at all stages and levels of representation of the *in silico* studies.
